# Comparison of 0.2% chlorhexidine mouthwash with and without anti-discoloration system in patients with chronic periodontitis: A randomized controlled clinical trial

**DOI:** 10.15171/japid.2019.011

**Published:** 2019-12-18

**Authors:** Siamak Yaghobee, Farid Abedin Dorkoosh, Farnaz Kouhestani, Ghazaleh Mozafari, Hoori Aslroosta

**Affiliations:** ^1^Department of Periodontics and Dental Implant Research Center, School of Dentistry, Tehran University of Medical Sciences, Tehran, Iran; ^2^Department of Pharmaceutics, Faculty of Pharmacy, Tehran University of Medical Sciences, Tehran, Iran; ^3^Department of Periodontics, School of Dentistry, Shiraz University of Medical Sciences, Shiraz, Iran

**Keywords:** Anti-discoloration system, bleeding on probing, chlorhexidine, mouthwash, periodontitis, plaque control, staining

## Abstract

**Background:**

Chemical plaque control, an adjunct to mechanical approaches, could improve the maintenance of patients with different types of periodontitis. Chlorhexidine, the gold standard in chemical plaque control, might have some side effects; the most determining one is tooth discoloration. Anti-discoloration systems (ADS) have been added to minimize brownish tooth discoloration. This study aimed to evaluate the staining potential and clinical efficacy of chlorhexidine with and without ADS in patients with chronic periodontitis.

**Methods:**

In this randomized controlled trial, 46 patients with chronic periodontitis were randomly allocated to two groups. Each patient used 10 mL of mouthwash A (CHX without ADS) or B (CHX with ADS, including sodium metabisulfite and ascorbic acid) twice a day for two weeks. After a two-week interval, they used the second mouthwash. At the beginning and the end of each two-week cycle, plaque index (PI), bleeding on probing (BoP), and staining index were recorded.

**Results:**

There was no significant difference between mouthwash A and B in the reduction of BoP and PI. The staining index was significantly lower after rinsing with mouthwash B compared to mouthwash A.

**Conclusion:**

CHX mouthwash containing ADS has similar efficacy in microbial plaque control and reduction of BOP as CHX without ADS, with the advantage of lower stain formation on tooth surfaces in patients with chronic periodontitis.

## Introduction

Chlorhexidine is the most efficient chemical agent against oral biofilms. It has been studied as an effective agent with plaque inhibitory effect and anti-gingivitis activity.^1^ Chlorhexidine has been known as the gold standard in plaque control for over 40 years.^2,3^ Unfortunately, due to some side effects, long-term use of chlorhexidine mouthwash is not recommended. These side effects are mainly tooth discoloration and taste alteration, impairing patients’ compliance. Other less common side effects include oral mucosal erosion, unilateral or bilateral parotid swelling, enhancement of supragingival calculus formation, and difficulty in masking its bitter taste.^4^ The brownish discoloration of the teeth and tongue is due to the reduction of disulfide to thiol that forms dark complexes with iron (III) ions in the saliva. Other discolorations might be caused by monosaccharides, such as glucose and fructose, which are dissolved in saliva and react with the amine groups of bacterial proteins (Maillard reaction).^5-8^ Furthermore, the interaction of chlorhexidine molecules with dietary anionic chromogens can lead to brownish staining.^8^

Since patient compliance is strongly correlated with these side effects, different formulations of lower concentrations of CHX (0.05%) or products containing peroxoborate, polyvinyl pyrrolidone, sodium metabisulfite, and ascorbic acid (anti-discoloration system: ADS) have been launched to minimize tooth discoloration. The ADS seems to be more effective in reducing brownish stain formation.^9^

Conflicting results have been found regarding the effects of CHX containing ADS on clinical gingival indices, biofilm accumulation, and tooth discoloration.^10-13^ Most of the studies investigating the clinical efficacy have focused on experimental gingivitis. On the other hand, the positive effects of CHX administration have been demonstrated in patients with chronic periodontitis.^14,15^ This clinical study was designed to evaluate the extent of tooth discoloration developed by 0.2% CHX mouthwash containing ADS and its efficacy in reducing dental plaque and gingival inflammation compared to 0.2% CHX without ADS in patients with chronic periodontitis.

## Methods

### Study Design

This double-blind, comparative, randomized cross-over clinical trial, conducted in the Department of Periodontics, Dental School of Tehran University of Medical Sciences. The study was conducted under the Helsinki Declaration, as revised in 2000.

### Participants

The participants were selected from the patients referred to the Department of Periodontics, Tehran University of Medical Sciences, from September 2015 to November 2015. A thorough intraoral assessment was performed during the initial visit, including full-mouth periodontal probing and measurement of clinical attachment level. Radiographic evaluation was performed on panoramic radiographs; 125 patients were diagnosed with chronic periodontitis. Patients with the following criteria were excluded:

1) Suffering from uncontrolled or poorly controlled diabetes, unstable or life-threatening conditions or use of antibiotics within the last six months

2) Smoking

3) Known allergy to CHX

4) Stain on teeth that could not be removed by polishing.

The participants underwent supragingival scaling, subgingival scaling, and prophylaxis. They were given instructions in oral hygiene. Following manual toothbrushing, the patients were instructed on the use of an interdental brush and/or dental floss with appropriate size. They were instructed to use the toothbrush with a 30-minute interval between brushing and using the mouthwash to avoid any interaction between anionic compounds in the toothpaste and chlorhexidine. After three months of follow-up, 64 patients who achieved PI≤25%^10^ were given detailed information about the study design, and a written consent form was obtained. Eighteen patients were excluded because they could not follow the study procedures.

The other 46 patients with chronic periodontitis were randomly divided into two groups, with 23 patients in each (groups 1 and 2). Randomization was performed by one of the authors who was not directly involved in the treatment of the patients. A computer-generated random sequence was used to assign patients to one of the two groups. The allocation was concealed using a code to identify the allocated group. It was sealed in an opaque pocket and was opened after the clinical examination.

### Pre-treatment and Treatment Phase

The mouthwash samples for the study were previously labeled as A (0.2% CHX without ADS; Iran Najo Co., Tehran, Iran) and B (0.2% CHX with ADS; Curasept; Curaden Healthcare Srl, Saronno, Italy). The ADS ingredients in the latter mouthwash were sodium metabisulfite and ascorbic acid. In order to ensure double-blinding, the bottles containing both types of mouthwash were the same with no difference. Therefore, neither the patient nor the clinician was able to recognize the type of mouthwash based on their bottle. Also, labeling the mouthwash was masked from the clinicians.

Immediately before starting treatment with the mouthwash, full-mouth plaque score (FMPS) and full-mouth bleeding score (FMBS) were recorded for all the participants. All the measurements were performed using a UNC-15 periodontal probe by the same calibrated and blinded expert. The first group (group 1) was instructed to use mouthwash A for two weeks, twice a day, and 10 mL each time. After this period, the mentioned indices were re-evaluated, and the staining index was recorded. In the following two weeks, the patients did not use any mouthwashes (wash-out period). After that, they were recalled and underwent full-mouth prophylaxis. Then, the clinical parameters were measured again. For the next two weeks, they used the second mouthwash (mouthwash B) in the same method. At the end of this period, the clinical measurements and prophylaxis were repeated for the last time. The second group (group 2) underwent the same process, but they first received mouthwash B and then mouthwash A. All the participants were asked to fill out a diary and to bring the empty mouthwash bottles to show their compliance.

### Clinical Measures

The full mouth plaque score (FMPS) was recorded according to the O’Leary index.^13^ A periodontal probe was used to define a binary score for each surface based on the presence or absence of microbial plaque. FMPS was scored after calculating the percentage of surfaces presenting microbial plaque. Gingival index (GI) was recorded on four surfaces (Loe, 1967) of each tooth; then, the mean was calculated for each patient.

**Figure 1:**
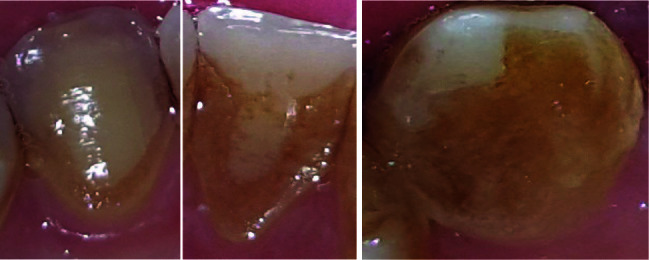
Staining index. a) score 1, b) score 2, c) score 3. The images were taken with an intraoral camera.

The staining index (SI) was assessed on the buccal and lingual/palatal surfaces of all the teeth using the Modified Lobene Staining Index.^16^ Each tooth surface was divided into gingival, incisal, and proximal areas. The intensity of staining in each region was scored as follows (Figure 1):

Score 0: no staining

Score 1: less than 1/3 of the area was covered with stain

Score 2: 1/3‒2/3 of the area was covered with stain

Score 3: more than 2/3 of the area was covered with stain.

### Data Analysis

Wilcoxon signed-rank test was used to analyze the data related to FMBS, SI, and FMPS between the treatment groups at baseline and after two weeks of mouthwash use.

Repeated-measures ANOVA was used for the analysis of the data related to probing depth at a significance level of P<0.05.

## Results

**Figure 2:**
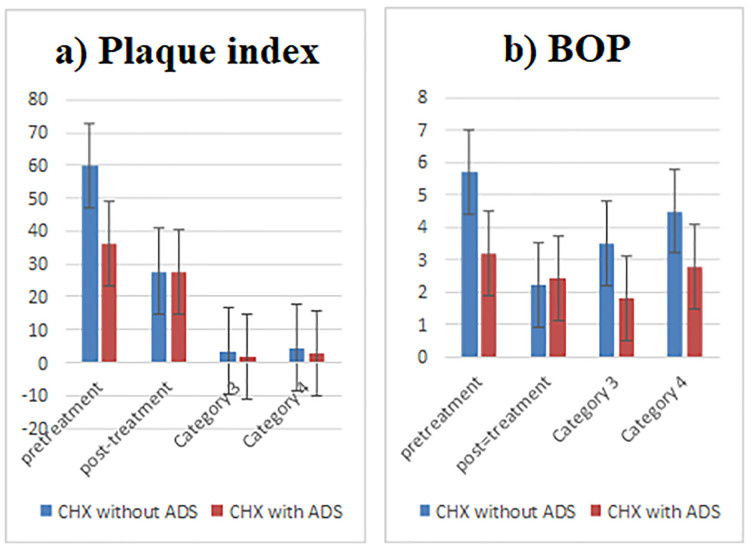
Comparison of means ± SD of a) plaque index and b) BOP at baseline and 2 weeks after treatment.

All 46 patients completed both treatment phases. No complications or adverse effects were reported. At baseline, no significant difference was found in FMPS, GI, and PI between the two groups (P=0.424, P=0.341, and P=0.712, respectively) (Table 1, Figure 2). Intra-group comparison in both groups showed a significant decrease in FMPS, GI, and PI values in the follow-up visits (P<0.05) (Table 2). The follow-up results demonstrated no significant difference in GI and FMPS between the two types of mouthwash. Both types of mouthwash showed similar effectiveness in plaque reduction and improvements in gingival inflammation without a significant difference (P=0.815 and P=0.501, respectively).

**Table 1:** Inter-group comparison of pre-treatment and post-treatment levels of GI, PI and FMBS

	**Mouthwash A**	**Mouthwash B**	**P-value**
**GI (before treatment)**	4.212±1.323	4.153±1.652	0.412 (NS)
**GI (after treatment)**	2.103±1.112	2.762±1.013	0.102 (NS)
**PI (before treatment)**	35.210±12.02	36.152±12.95	0.541 (NS)
**PI (after treatment)**	27.739±13.04	27.478±12.9	0.618 (NS)
**FMBS (before treatment)**	3.782±1.107	3.195±1.328	0.814 (NS)
**FMBS (after treatment)**	2.239±1.302	2.413±1.514	0.201 (NS)

**Table 2:** Intra-group comparisons (pre- and post-treatment)

	**Before treatment**	**After treatment**	**P-value**
**Mouthwash A**			
**GI**	4.212±1.323	2.103±1.112	0.021
**PI**	35.210±12.02	27.739±13.04	0.014
**FMBS**	3.782±1.107	2.239±1.302	0.034
**Mouthwash B**
**GI**	4.153±1.652	2.762±1.013	0.032
**PI**	36.152±12.95	27.478±12.9	0.010
**FMBS**	3.195±1.328	2.413±1.514	0.029

Regarding post-treatment BoP, no significant difference was found between the two types of mouthwash (P=0.501).

**Figure 3:**
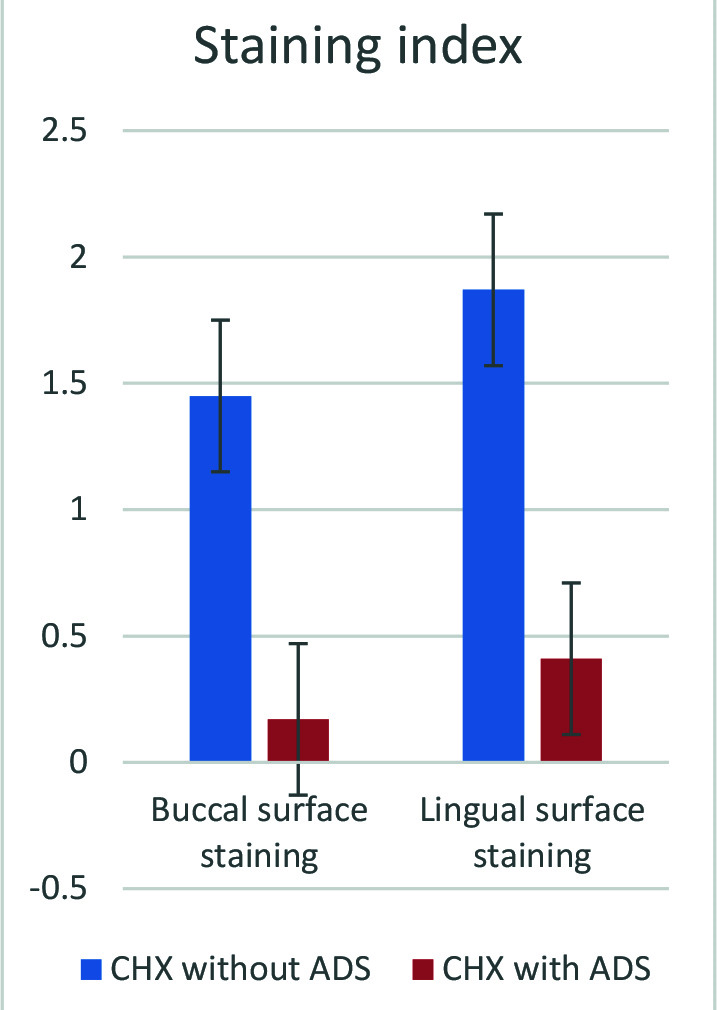
Buccal and lingual staining index two weeks after treatment.

A higher deposition of extrinsic stain was detected with mouthwash A (Figure 3). The CHX mouthwash containing ADS caused a significant decrease in tooth discoloration compared to the CHX without ADS (P<0.001).

## Discussion

This study was conducted to compare the efficacy of 0.2% CHX mouthwash containing ADS and the conventional CHX mouthwash in controlling plaque and gingivitis. Furthermore, the extent of tooth discoloration caused by these two types of mouthwash was examined. The results suggested no statistically significant differences between these two types of mouthwash for their power in decreasing plaque formation and gingival inflammation. However, the stain formation score was significantly higher in group A as compared to group B. One of the major concerns of the patients with the use of CHX mouthwash is tooth discoloration, which results in unaesthetic appearance and could cause social embarrassment.^17^ Therefore, the patient might not cooperate well and restrict the use of the mouthwash. The results of the current study showed that patients could use CHX mouthwash containing ADS without any worries about its efficacy and with less tooth discoloration.

In a study by Bernardi et al,^9^ periodontally healthy volunteers with experimental gingivitis were given 0.2% ADS CHX mouthwash. They compared this mouthwash with 0.2% CHX mouthwash. The participants of this study used each mouthwash for 15 days with a 15-day wash-out period. The researchers evaluated the PI and GI and assessed staining caused by these two types of mouthwash. They reported no significant difference for PI and GI between the two types of mouthwash in healthy patients. However, a statistically significant difference was observed in the extent of staining. The researchers recommended the need to conduct this

study in patients with periodontitis since the exposed rough cementum surfaces in these patients are more susceptible to staining. In 2011, a similar study with the same sample size was carried out by Solis et al^18^ except that in their study, they evaluated patients with chronic periodontitis. These researchers also reported the same results as Bernardi et al.^9^ Solis et al^18^ pointed out the small size of the sample was a limitation of their study and recommended further clinical studies on patients with periodontitis. In 2008, Cortellini et al^10^ used 0.2% CHX with ADS system and 0.2% CHX mouthwash in 48 patients for one week after periodontal surgery. The clinicians did not allow any dental or interdental brushing over the area that underwent surgery. One week later, after suture removal, full professional prophylaxis was performed, and the second mouthwash was given with the same indications of use as the first one. They also observed less staining with the use of 0.2% CHX mouthwash with ADS. However, they reported the two types of mouthwash had similar effects concerning the reduction of gingival inflammation after surgery.

These findings are consistent with the results of the present study. However, some other studies have reported results different from those of the present study. In 2006, Arweiler et al^19^ designed a 4-day plaque re-growth study to compare the efficacy of two CHX types of mouthwash with and without ADS. Twenty-one volunteers, without using any brushing, rinsed twice a day with a regular CHX mouthwash, or a CHX mouthwash containing ADS, or a placebo. The plaque index and plaque areas were assessed after 24 and 96 hours. They reported that although ADS resulted in stain reduction, it decreased the effectiveness of CHX on dental plaque reduction. In 2012, Li et al^11^ designed an experimental gingivitis model study to evaluate the anti-gingivitis effect of chlorhexidine mouthwash with or without ADS. This study was conducted on 26 volunteers with a healthy periodontium. During three weeks of the experimental period, the participants did not use any mechanical oral hygiene means and used only mouthwashes. The discoloration, plaque, and gingival indices were assessed on days 0, 7, and 21. They concluded that CHX with ADS appeared to be useful in the prevention of staining but at the expense of some loss of anti-gingivitis and plaque control ability.

The diversity of results might arise from using or not using mechanical plaque control means, which can mask the real efficacy of mouthwashes. However, although in the study by Cortellini et al^10^ the patients were not allowed to brush and use interdental floss in the areas of surgery for the first week, the measurement of clinical indices in these areas did not agree with the findings reported by Li et al.^11^ The shorter period of assessment in the study by Cortellini et al^10^ can account for the difference. The difference between the results reported by Cortellini et al and Arweiler et al^19^ might be due to the differences in the choice of the study indices. While plaque seems to be a required prerequisite for gingival inflammation, a plaque-regrowth study with healthy volunteers does not allow conclusions on the effect of a mouthrinse on the gingival health of patients suffering from periodontitis.

The only study which did not approve the ability of ADS to reduce staining was carried out by Addy et al.^20^ The explanation for this difference would be that these researchers considered one of the CHX staining mechanisms (anionic dietary chromogens), while there are also other mechanisms as explained before. ADS seems to reduce staining by affecting the other mechanisms. On the other hand, some researchers argue that this anti-discoloration system does not influence the efficacy of CHX in plaque control and gingival inflammation reduction. They suggest that the efficacy of this mouthwash is not reduced, considering its substantivity in the oral cavity, and its incorporation into the bacterial membrane is not impaired. In fact, efforts to prevent the formation of brownish discoloration by the incorporation of reducing agents, such as ascorbic acid that react with iron (III) ions, and nucleophiles, such as sulfite ions that react with glucose and fructose, have been successful. The neutral ascorbic acid or the negatively charged ascorbate or the negatively charged sulfite does not affect the attachment of the two-fold positively charged CHX to the teeth and gingiva. Also, negatively charged sulfite or ascorbate and positively charged CHX are not combined to form a precipitate of CHX‒sulfite or CHX‒ascorbate. Such a combination would lead to a complete inactivation, and this has never been observed in the literature.

One of the limitations of the current study was that only tooth discoloration following the use of CHX mouthwash was evaluated. CHX mouthwash can affect not only the teeth but also the oral mucosa and any restoration in the oral cavity.^21^ However, in the current study, only discoloration of the teeth was evaluated as it can be considered as one of the most critical concerns of patients.

## Conclusion

According to the present study, the anti-discoloration system composed of ascorbic acid and sodium metabisulfite, resulted in less discoloration by CHX mouthwash, while it did not affect its efficacy in plaque and gingivitis control.

## Conflict of Interests

The authors declare no conflict(s) of interest related to the publication of this work.

## Authors’ Contributions

S.Y. conception and design of the study, technical support, supervision and approval of the final version of the manuscript. F.A.D study design, preparation of the test and control moutheashes. F.K. statistical analysis, interpretation of data. G.M. patient selection, performing the phase I periodontal treatment and following all participants for their adherence to the study design. H.A. data acquisition, interpretation of data, manuscript drafting and approval of the final version of the manuscript.

## Ethics Approval

Study protocol was approved by ethical committee of the school of dentistry, Tehran university of Medical Sciences.

## References

[1] Prasad KA, John S, Deepika V, Dwijendra KS, Reddy BR, Chincholi S (2015). Anti-Plaque Efficacy of Herbal and 02% Chlorhexidine Gluconate Mouthwash: A Comparative Study. J Int Oral Health.

[2] Jones CG (1997). Chlorhexidine: is it still the gold standard?. Periodontol 2000.

[3] Loe H, Schiott CR (1970). The effect of mouthrinses and topical application of chlorhexidine on the development of dental plaque and gingivitis in man. J Periodontal Res.

[4] Flotra L, Gjermo P, Rolla G, Waerhaug J (1971). Side effects of chlorhexidine mouth washes. Scand J Dent Res.

[5] Gilbert HF (1990). Molecular and cellular aspects of thiol-disulfide exchange. Adv Enzymol Relat Areas Mol Biol.

[6] Grandhee SK, Monnier VM (1991). Mechanism of formation of the Maillard protein cross-link pentosidine Glucose, fructose, and ascorbate as pentosidine precursors. J Biol Chem.

[7] Hjeljord LG, Rolla G, Bonesvoll P (1973). Chlorhexidine-protein interactions. J Periodontal Res Suppl.

[8] Addy M, Moran J, Griffiths AA, Wills-Wood NJ (1985). Extrinsic tooth discoloration by metals and chlorhexidine I Surface protein denaturation or dietary precipitation?. Br Dent J.

[9] Bernardi F, Pincelli MR, Carloni S, Gatto MR, Montebugnoli L (2004). Chlorhexidine with an Anti-discoloration System A comparative study. Int J Dent Hyg.

[10] Cortellini P, Labriola A, Zambelli R, Prato GP, Nieri M, Tonetti MS (2008). Chlorhexidine with an anti-discoloration system after periodontal flap surgery: a cross-over, randomized, triple-blind clinical trial. J Clin Periodontol.

[11] Li W, Wang RE, Finger M, Lang NP (2014). Evaluation of the antigingivitis effect of a chlorhexidine mouthwash with or without an antidiscoloration system compared to placebo during experimental gingivitis. J Investig Clin Dent.

[12] Grundemann LJ, Timmerman MF, Ijzerman Y, van der Weijden GA, van der Weijden GA (2000). Stain, plaque and gingivitis reduction by combining chlorhexidine and peroxyborate. J Clin Periodontol.

[13] Graziani F, Gabriele M, D'Aiuto F, Suvan J, Tonelli M, Cei S (2015). Dental plaque, gingival inflammation and tooth -discolouration with different commercial -formulations of 02% chlorhexidine rinse: a double-blind randomised controlled clinical trial. Oral Health Prev Dent.

[14] Fonseca DC, Cortelli JR, Cortelli SC, Miranda Cota LO, Machado Costa LC, Moreira Castro MV (2015). Clinical and Microbiologic Evaluation of Scaling and Root Planing per Quadrant and One-Stage Full-Mouth Disinfection Associated With Azithromycin or Chlorhexidine: A Clinical Randomized Controlled Trial. J Periodontol.

[15] da Costa L, Amaral C, Barbirato DDS, Leao ATT, Fogacci MF (2017). Chlorhexidine mouthwash as an adjunct to mechanical therapy in chronic periodontitis: A meta-analysis. J Am Dent Assoc.

[16] Akwagyiram I, Butler A, Maclure R, Colgan P, Yan N, Bosma ML (2016). A randomised clinical trial to evaluate the effect of a 67 % sodium bicarbonate-containing dentifrice on 02 % chlorhexidine digluconate mouthwash tooth staining. BMC Oral Health.

[17] Marrelli M, Amantea M, Tatullo M (2015). A comparative, randomized, controlled study on clinical efficacy and dental staining reduction of a mouthwash containing Chlorhexidine 020% and Anti Discoloration System (ADS). Ann Stomatol (Roma).

[18] Solis C, Santos A, Nart J, Violant D (2011). 0.2% chlorhexidine mouthwash with an antidiscoloration system versus 02% chlorhexidine mouthwash: a prospective clinical comparative study. J Periodontol.

[19] Arweiler NB, Boehnke N, Sculean A, Hellwig E, Auschill TM (2006). Differences in efficacy of two commercial 02% chlorhexidine mouthrinse solutions: a 4-day plaque re-growth study. J Clin Periodontol.

[20] Addy M, Sharif N, Moran J (2005). A non-staining chlorhexidine mouthwash? Probably not: a study in vitro. Int J Dent Hyg.

[21] Moran JM (2008). Home-use oral hygiene products: mouthrinses. Periodontol 2000.

